# Real World Effectiveness of Information and Communication Technologies in Disaster Relief: A Systematic Review

**DOI:** 10.18502/ijph.v49i10.4678

**Published:** 2020-10

**Authors:** Bingqing LU, Xingyi ZHANG, Jin WEN

**Affiliations:** 1. Chinese Evidence-Based Medicine Center, West China Hospital, Sichuan University, Chengdu 610041, China; 2. Department of Information Engineering, School of Telecommunications Engineering, Xidian University, Xi’an 710071, China; 3. Institute of Hospital Management, West China Hospital, Sichuan University, Chengdu 610041, China

**Keywords:** Information and communication technologies (ICTs), Natural disaster, Disaster relief

## Abstract

**Background:**

The application of information and communication technologies (ICTs) in disaster relief is increasingly widespread, but it is still unclear whether ICT can reduce casualties and economic losses in disaster response phase.

**Methods:**

We searched studies in the databases of Scopus, EI, MEDLINE and EMBASE from Jan 1, 1990, to Mar 22, 2019. Excel 2016 and VOSviewer (version 1.6.11) were used to analyze the extracted data and visualize the network diagram.

**Results:**

We included 169 eligible articles. The number of ICTs-related disaster-relief articles published annually shows an overall trend of growth since 1990. The United States has the greatest influence in this field. The 169 articles reported twenty-four technologies and the top three reported most frequently were remote sensing, social media, and geographic information system (GIS). The main roles of ICTs in natural disaster rescue included information dissemination, post-disaster image collection and damage assessment. However, of the 169 articles, only five reported that ICTs reduced casualties or economic losses in disaster response phase, two concluded that rescue robot was ineffective in mudslide rescue, and the remaining 162 (95.86%) did not evaluate the effect of ICTs on the rescue.

**Conclusion:**

ICTs have the potential to reduce casualties and economic losses, but some technologies are not applicable to all rescue scenarios. In addition, most studies did not pay attention to the effect of technology on the rescue.

## Introduction

### Natural disasters

Natural disasters, including earthquakes, floods and tsunamis, have become one of the most serious threats to human health and property. On the one hand, the frequency of natural disasters has increased due to environmental deterioration. The increase in population density and the disordered growth of urban areas have increased the number of people affected by disasters (1, 2). From 1990 to 2018, 9426 recorded natural disasters affected over 5.89 billion people, killing an additional 1.65 million and costing a total of US $2.95 trillion (3).

### Disaster Management

In order to reduce the adverse consequences of disasters, the field of disaster management emerged (4). Disaster management is the systematic process of using administrative decisions, organization, operational skills and capacities to implement policies, strategies and coping capacities of the society and communities to lessen the impacts of disasters (5). Usually, the disaster management period is divided into four stages: prevention/mitigation, preparedness, response and recovery, which form a cycle (6, 7). Initially, prevention/mitigation refers to taking actions to prevent or mitigate the impact of disasters before they occur. Such as building dams, identifying risk areas and disaster education; next, preparedness is the activities that develop operational capabilities for responding to disasters. It includes establishing an early warning system, developing a disaster response plan; Then response refers to taking rescue measures to reduce casualties and economic losses during or after disasters; Finally, post-disaster recovery focuses on mobilizing resources, stabilizing and rebuilding infrastructure in the affected areas, and restoring normal life for the victims (5,7–10). In recent decades, with the rise of the Internet, information and communication technologies (ICTs) have played a critical role in all phases of disaster management, especially in the response phase (11). However, the current rescue process is mainly based on experience and there is a lack of research on the best practices for ICTs deployment in this phase (11). Therefore, for further research, it is necessary to fully understand the current application of ICTs in rescue.

Information and communication technology (ICT) as a diverse set of technological tools and resources were defined that can be used to communicate, create, disseminate, store and manage information and knowledge in the global context (12). In 2002, UNESCO acknowledged that ICTs include some technologies applied in the collection, storage, editing, retrieval and transfer of information in various forms, which can be divided into telecommunication technologies, digital technologies and software applications (13). ICT is considered an extended synonym for information technology to emphasize the integration of the unified (tele) communications (14). Similarly, information technology (IT) is defined as “any technology used to support information gathering, processing, distribution, and use and is composed of hardware, software, data, and communication technology” (15). Telecommunications is the transmission of symbols, signals, messages, words, images, sounds or information of any nature through wired, radio, optical or electromagnetic systems (16). According to Sallai ‘s definition of ICT, in this study, ICTs refer to all information technologies including remote sensing, geographic information system (GIS), global positioning system (GPS), radar, radio, email, telephone, short message, video, information system, Internet, website, social media, online forum, telemedicine, unmanned aircraft system (UAS), etc.

The objective of this study was to sort out the published articles on ICTs and natural disaster rescue through systematic review, to understand the application status and effect of ICTs in disaster response, and provide evidence for the research on rescue effect evaluation of ICTs and optimizing ICTs deployment in relief.

## Methods

This study adopted the method of systematic review to systematically search, screen and synthesize the extracted data for articles on the application of ICTs in natural disaster rescue. We followed the PRISMA statement for the reporting of this systematic review (17). No protocol for this systematic review existed or was published beforehand. The PRISMA checklist was used.

### Search Strategies

The electronic databases (Scopus, EI, MEDLINE, EMBASE) were searched by two independent researchers from Jan 1, 1990, to Mar 22, 2019. The search terms used in Scopus and EI are shown in Table 1. Since MEDLINE and EMBASE only refer to biomedical literature, we used “telemedicine” as the representative of ICTs to search in the two databases (Table 2). The search terms were determined through literature review (18–25). At the same time, we limited the document type to “journal article” and the language to “English”.

**Table 1:** Search terms used in the databases of Scopus and EI

***Operator***	***Search fields and search terms***
AND	TITLE-ABS-KEY (disaster OR flood OR cyclone OR drought OR hurricane OR tornado OR typhoon OR wildfire OR earthquake OR tsunami OR volcano OR landslide OR avalanche OR rainstorm OR epidemic) TITLE-ABS-KEY (ICT OR “Information technolog^*^” OR “Communication technolog^*^” OR radio OR phone OR SMS OR “Short Message Service” OR “text messaging” OR media OR television OR e-mail OR internet OR website OR video OR teleconferencing OR “Instant Messenger” OR “Social Networking Site” OR SNS OR blog OR Instagram OR Facebook OR twitter OR myspace OR Flickr OR Sina-Weibo OR “online forum” OR GIS OR “Geographic Information System” OR “Geo-Information system” OR “geomatics technology” OR GPS OR “Global Positioning System” OR “remote sensing” OR radar OR “satellite sensor” OR “Airborne lidar” OR “emergency communication” OR “information management system” OR “decision support system” OR “knowledge management system” OR telemedicine OR “ad-hoc network” OR “wireless sensor network” OR “body area network” OR “image processing technology” OR “Internet of things” OR IoT OR UAS OR UAV OR “Unmanned aircraft” OR “Unmanned Aerial” OR “search and rescue robot” )
AND	TITLE-ABS-KEY (“Disaster management” OR “emergency management” OR “disaster response” OR “emergency response” OR rescue OR relief OR “disaster aid” OR “disaster assistance”)

**Table 2:** Search terms used in the databases of MEDLINE and EMBAS

***Operator ***	***Search fields and search terms***
	TITLE-ABS-KEY (disaster OR flood OR cyclone OR drought OR hurricane OR tornado OR typhoon OR wildfire OR earthquake OR tsunami OR volcano OR landslide OR avalanche OR rainstorm OR epidemic)
AND	TITLE-ABS-KEY (telemedicine)
AND	TITLE-ABS-KEY (“Disaster management” OR “emergency management” OR “disaster response” OR “emergency response” OR rescue OR relief OR “disaster aid” OR “disaster assistance”)

### Inclusion and Exclusion Criteria

All obtained articles were selected based on inclusion and exclusion criteria; articles that fully meet the following inclusion criteria were included:

- The research object of papers is natural disasters.
- Studies used ICTs in the disaster response phase.
- Articles analyzed the performance, function or influence of technology in rescue.

Articles that meet any of the following conditions were excluded:

- Review or serial (book series).
- The technology has not been used for actual rescue.

### Data Extraction

The data we extracted from eligible articles included article title, publication year, journal title, number of citations, corresponding authors as well as their countries and institutions, technology type, disaster type, disaster occurrence time, disaster area, landform of disaster area, time of technology application, and the role and effectiveness of technology in disaster relief.

### Related Definition

Effectiveness, if the use of ICTs in relief reduced casualties and economic losses, we judged that the technology was effective.

### Analysis Methods

Microsoft Excel 2016 software was used for descriptive statistical analysis of extracted data; VOSviewer software (version 1.6.11) was used for keywords co-occurrence network visualization and statistics of countries and institutions participating in the publication of included articles. VOSViewer developed by the Centre for Science and Technology Studies at Leiden University (The Netherlands), is a software tool specifically designed for constructing and visualizing bibliometric maps. It is able to analyze the files exported from Web of Science, PubMed, Scopus, and RIS format. In addition, VOSviewer accepts network data as well as textual data. Moreover, VOSviewer provides three visualizations of a map: the network visualization, the overlay visualization, and the density visualization (26). In the network visualization, the size of a node’s circle and label portray its importance. Larger circles and labels represent more important nodes. Further, the distance between two nodes in the map represents the strength of their relationship. The closer the distance is, the stronger the correlation is. Moreover, the color of the circles also depicts the cluster the node belongs to (27, 28).

### Quality Control

Two investigators independently completed the literature searches and screening, and disagreements were resolved by consensus as well as discussion. We used software Endnote for document management.

## Results

**Fig. 1:**
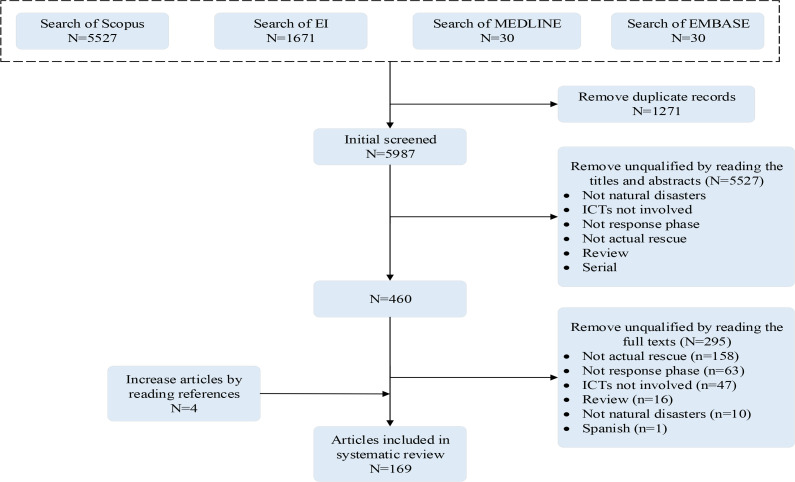
Flow diagram for searching and selecting study

A total of 7,258 references were returned through searching the databases of Scopus, EI, MEDLINE and EMBASE, of which 5987 were retained after removing the duplicate results using Endnote. Next, according to the inclusion and exclusion criteria, 460 potentially qualified records were identified by reading the titles and abstracts. Through further full-text assessment, 165 articles were eligible. Moreover, another four studies were included by reading references. Therefore, 169 articles were finally included in the systematic review (list of articles, see S2 Appendix). The flow chart for the search strategy was shown in Fig. 1.

### Number of Publications

**Fig. 2:**
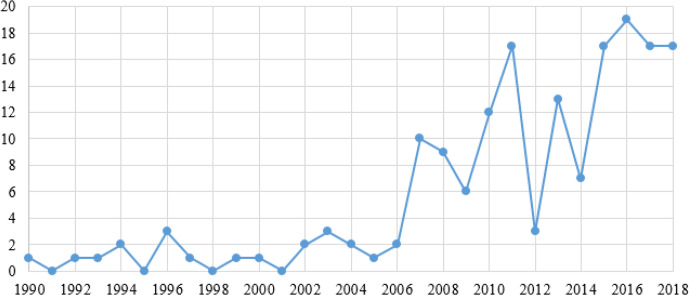
Number of publications on the application of information and communication technologies (ICTs) in disaster relief from 1990 to 2018

One hundred and sixty-nine articles were included in the systematic review through searching and screening based on specific search strategies and eligibility criteria. Of which 168 (99.4%) were published between 1990 and 2018. As can be seen from Fig. 2, the number of papers studying ICTs and actual disaster relief shows a general trend of growth since 1990. During 1990–2018, the number of annual publications was relatively stable. Further, there has been a significant increase since 2006, with two publishing peaks in 2011 and 2016.

### Journal Analysis

One hundred and sixty-nine articles included in the systematic review were published in one hundred and seventeen journals. Table 3 lists the related information of the ten active journals, including the number of publications, citations and 2017 IF (Impact Factor). International Journal of Remote Sensing (IF = 1.782, 2017) published the most studies with eight publications. Photogrammetric Engineering and Remote Sensing (IF = 3.15, 2017) and Natural Hazards (IF = 1.901, 2017) published seven articles respectively. In addition, PLoS ONE (IF= 2.766, 2017) is the most frequently cited journal with 1084 (28.23%) citations, an average of three hundred and sixty-one citations per document.

**Table 3:** Top 10 journals with most published articles on information and communication technologies (ICTs) and actual rescue during 1990–2019

***Rank***	***Journal title***	***No. of publications (%)***	***No. of citations (%)***	***2017 IF***
1	International Journal of Remote Sensing	8 (4.73)	241 (6.28)	1.782
2	Photogrammetric Engineering and Remote Sensing	7 (4.14)	188 (4.90)	3.15
2	Natural Hazards	7 (4.14)	123 (3.20)	1.901
3	Journal of Field Robotics	6 (3.55)	215 (5.60)	3.46
4	International Journal of Emergency Management	5 (2.96)	12 (0.31)	None
5	PLoS ONE	3 (1.78)	1084 (28.23)	2.766
5	Disasters	3 (1.78)	122 (3.18)	1.596
5	Journal of Homeland Security and Emergency Management	3 (1.78)	14 (0.36)	0.712
5	GEO: connexion	3 (1.78)	0 (0)	None
5	Journal of Emergency Management	3 (1.78)	12 (0.31)	None

### Country Analysis

The 169 articles included were contributed by fifty countries. There were ten countries publishing five or more papers (Table 4), and the United States had the first place when ranking for the number of publications (74, 43.79%), followed by China (20, 11.83%) and the United Kingdom (13, 7.69%). In addition, the United States was still ranked first in terms of citation number (2106, 54.84%), followed by Canada (607, 15.81%) and China (258, 6.72%).

**Table 4:** The top ten countries with the most papers on ICTs and actual rescue between 1990 and 2019

***Rank***	***Country***	***No. of publications (%) ^a^***	***No. of citations (%) ^a^***
1	United States	74 (43.79)	2106 (54.84)
2	China	20 (11.83)	258 (6.72)
3	United Kingdom	13 (7.69)	174 (4.53)
4	Germany	9 (5.33)	69 (1.80)
4	Italy	9 (5.33)	143 (3.72)
4	Australia	9 (5.33)	61 (1.59)
5	Canada	7 (4.14)	607 (15.81)
5	India	7 (4.14)	45 (1.17)
6	France	6 (3.55)	52 (1.35)
7	Japan	5 (2.96)	15 0.39)

a The total percentage of all countries is greater than 1, as cooperation existing between countries

### Institution Analysis

A total of 282 different organizations participated in the publication of the 169 articles on ICTs and natural disaster response for the period 1990– 2019. Table 5 shows the top nine active organizations in the field of ICTs and actual disaster relief. Out of the nine organizations, five were based in the United States. According to the number of articles, Chinese Academy of Sciences (5, 2.96%) was the most active institution followed by University of Colorado (4, 2.37%), and the other seven institutions (3, 1.78%). Moreover, the University of Colorado ranked first in terms of the number of citations (189, 4.92%), followed by the University of South Florida (146, 3.80%) and the Centers for Disease Control and Prevention (CDC) (129, 3.36%).

**Table 5:** The top nine institutions with the most papers on ICTs and actual rescue between 1990 and 2019

***Rank***	***Institution***	***Country***	***No. of publications (%) ^a^***	***No. of citations (%) ^a^***
1	Chinese Academy of Sciences	China	5 (2.96)	69 (1.80)
2	University of Colorado	United States	4 (2.37)	189 (4.92)
3	Georgia Institution of Technology	United States	3 (1.78)	21 (0.55)
3	National Aeronautics and Space Administration (NASA)	United States	3 (1.78)	124 (3.23)
3	University of South Florida	United States	3 (1.78)	146 (3.80)
3	Centers for Disease Control and Prevention (CDC)	United States	3 (1.78)	129 (3.36)
3	India Institution of Technology	India	3 (1.78)	28 (0.73)
3	University of Cambridge	United Kingdom	3 (1.78)	21 (0.55)
3	University of Southampton	United Kingdom	3 (1.78)	23 (0.60)

a The total percentage of all institutions is greater than 1, as cooperation existing between institutions

### Keywords Analysis

**Fig. 3:**
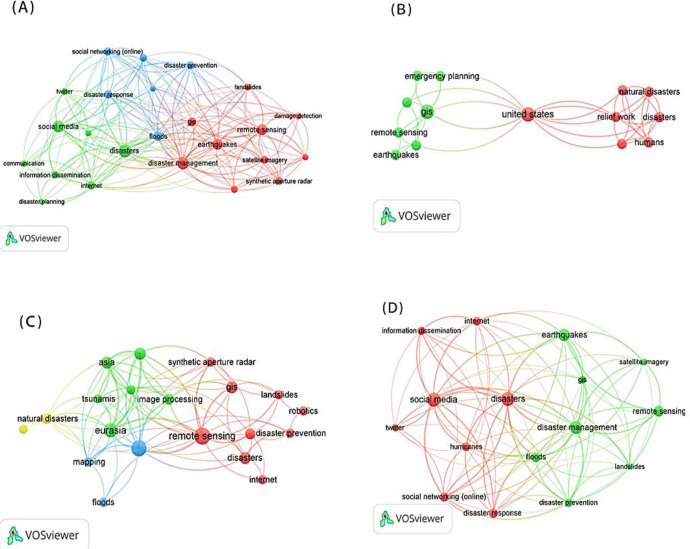
The keywords co-occurrence network of publications on ICTs and actual rescue. (A) Shows the keywords with high frequency of occurrence in the papers published between 1990 and 2019. (B), (C) and (D) respectively show the keyword co-occurrence networks in the three time periods of 1990–1999, 2000–2009 and 2010–2019

Keywords co-occurrence network helps to discover research hotspots and directions (29). Fig. 3 (A) indicates the keywords co-occurrence network of the articles on ICTs and actual rescue during 1990–2019. The top ten keywords with the highest frequency in the articles included in the past thirty years were disaster management, disasters, social media, earthquakes, remote sensing, humans, floods, GIS, disaster response and Internet. In order to further explore the research trend in this field, the thirty years were divided into three decades, namely 1990–1999, 2000– 2009 and 2010–2019. Figure 3 (B), (C) and (D) show the high frequency keywords in the three time periods respectively. As shown in Fig. 3 (B), in the 1990s, the hot keywords in the field of ICTs and natural disaster response included GIS, remote sensing, aerial photography, and telemedicine. At the beginning of the 21st century, popular technologies applied to disaster response include remote sensing, GIS, image processing, synthetic aperture radar, Internet and robotics (Fig. 3 C). Afterwards, in the past ten years, social media (such as Twitter) and social networking (online) have become new research hotspots in this field. However, traditional ICTs such as remote sensing and GIS have still appeared at a relatively high frequency in the papers of the past decade (Fig. 3 D).

### Types of Natural Disasters

**Fig. 4:**
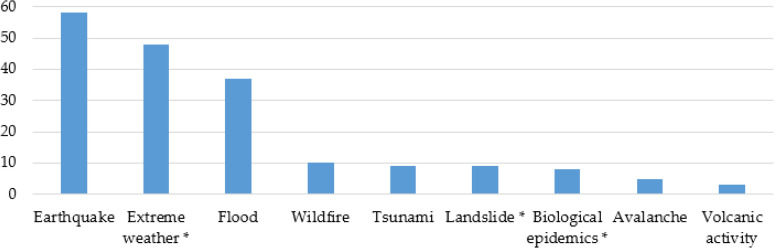
The nine kinds of natural disasters researched by the 169 articles. In this study extreme weather includes hurricane, tornado, typhoon, storm, cyclone, blizzard and rainstorm. Landslide includes landslide and mudslide. Then biological epidemics include H1N1, H7N9, Ebola, Zika virus and SARS outbreak

A total nine kinds of natural disasters were studied by the 169 studies included in the systematic review, including earthquake, extreme weather, flood, wildfire, tsunami, landslide, biological epidemics, avalanche and volcanic activity (Fig. 4). Earthquake was the most studied natural disaster (58, 34.32%), followed by extreme weather (48, 28.40%) and flood (37, 21.89%).

### Top seven Natural Disaster Events Most Studied

Of the 169 articles included, the most frequently studied natural disaster event was the 2010 Haiti earthquake (19,11.24%). The number of articles on 2005 Hurricane Katrina and 2008 Sichuan earthquake was 13 (7.69%) and 10 (5.92%) respectively, ranking second and third. Table 6 lists the top seven natural disaster events.

**Table 6:** Analysis of the characteristics of disasters

***Categories***	***No. of publications (%)***
Top seven disaster events	
2010 Haiti earthquake	19 (11.24)
2005 Hurricane Katrina	13 (7.69)
2008 Sichuan earthquake	10 (5.92)
2013 Typhoon Haiyan	8 (4.73)
2004 Indian Ocean tsunami	8 (4.73)
2012 Hurricane Sandy	6 (3.55)
April 2015 Nepal earthquake	5 (2.96)
Landform of disaster areas ^a^	
Plain	87 (51.48)
Mountain area/hills	72 (42.60)
Complex terrain	21 (12.43)
Basin	5 (2.96)
Plateau	2 (1.18)

a The total percentage is greater than 1, as some papers studied more than one disaster events

### Landform of Disaster Areas

Table 6 also shows the landform of the disaster areas researched by the 169 papers included, including plain, mountain area/hills, basin and plateau. Among them, plain and mountain area were the most involved, accounting for 51.48 % (n=87) and 41.60 % (n=72) respectively. Basin and plateau were less involved, accounting for 2.96 % (n=5) and 1.18 % (n=2) respectively. Another twenty-one articles reported that the rescue took place on the compound terrain.

### Types of ICTs

**Fig. 5:**
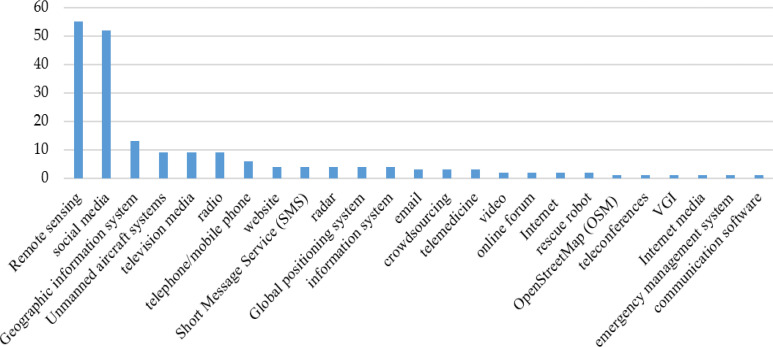
The twenty-four kinds of ICTs involved in the publications on ICTs and natural disaster response during 1990–2019. VGI in the figure is an abbreviation of volunteered geographic information

A total of twenty-four kinds of ICTs were applied in the 169 studies (Fig. 5). Judging from the number of articles, remote sensing is the most studied technology (55, 32.54%), followed by social media (52, 30.77%) and GIS (13, 7.69%).

### Time of ICTs Application

Of the 169 studies, only 72 (42.60%) reported the time of ICTs application in disaster relief. There were fifty-two papers reported that ICTs were applied within 72 hours after the disaster. More detailed information on ICTs application time is shown in Table 7.

**Table 7:** Analysis of the characteristics of ICTs

***Categories ***	***No. of publications (%)***
Time of ICTs application in rescue	
≤6h	14 (8.28)
≤24h	16 (9.47)
≤48h	13 (7.69)
≤72h	9 (5.33)
>72h	20 (11.83)
Unclear	97 (57.40)
Role of ICTs in rescue ^a^	
Dissemination of disaster-related information	82 (48.52)
Image collection of disaster areas	62 (36.69)
Damage assessment	40 (23.67)
Coordination of rescue work	19 (11.24)
Donations collection	19 (11.24)
Geo-information display and analysis	17 (10.06)
Emotional support	9 (5.33)
Volunteer mobilization	6 (3.55)
Looking for missing	6 (3.55)
Data management	5 (2.96)
Location of affected areas	4 (2.37)
Location the victims	4 (2.37)
Provision of telemedicine	3 (1.78)
Effectiveness	
Effective	5 (2.96)
Ineffective	2 (1.18)
Unclear	162(95.86)

a The total percentage is greater than 1, as some articles studied the multiple roles of ICTs

### Role of ICTs in Rescue

Table 7 displays the role of ICTs in actual disaster relief according to the articles included, mainly including information dissemination, image collection, damage assessment, coordination of rescue work, donations collection, and geoinformation display and analysis. Eighty-two (48.52%) articles reported that ICTs can disseminate disaster-related information during the disaster response phase. Sixty-two (36.69%) papers reported that remote sensing and other technologies can collect images of disaster areas for disaster assessment.

### Effectiveness of ICTs in Rescue

Table 7 also lists the distribution of technical effectiveness in 169 articles. Only five of these articles made it clear that ICTs was effective for rescue, that is, it can reduce casualties or economic losses (30–34). Two other articles concluded that the rescue robot was ineffective in the mudslide rescue because of its poor water resistance (35, 36). Furthermore, the majority failed to evaluate the effect of ICTs on the rescue.

## Discussion

We conducted a review of the articles on the effectiveness of ICTs in natural disaster relief in the databases of Scopus, EI, MEDLINE and EMBASE from Jan 1, 1990, to Mar 22, 2019. A total of 169 studies that met the inclusion and exclusion criteria were included. Through this review, we can find ICTs are more and more widely used in natural disaster relief, and it plays a vital role in information dissemination, providing disaster information, assessing disaster damage, coordinating rescue and financing. ICTs have the potential to reduce casualties and economic losses in the disaster response phase, while some technologies are not yet applicable to all rescue scenarios, and most articles did not evaluate the effect of ICTs on the rescue.

The keywords co-occurrence network indicates that since 1990, remote sensing technology and GIS have played a significant role in disaster relief (Fig. 3). Remote sensing technology is often employed in conjunction with image processing technology or/and GIS technology. When a disaster occurs, remote sensing technology (mainly satellite remote sensing) is used to acquire images of disaster areas, the image processing technology is used to process the images, and the damaged images of disaster areas can be displayed on a three-dimensional map through GIS technology (37, 38). Satellite remote sensing has the characteristics of large space coverage and high cost-effectiveness (39), but at the same time it may be limited by weather and its own operation cycle (40). In this case, it is often necessary to employ aerial remote sensing, ground detection, and satellite remote sensing to collect all-round information in disaster areas (41). The information can help assess the extent and distribution of damages in disaster areas, plan rescue routes, and distribute rescue materials (42, 43).

As can also be seen from the keywords cooccurrence network (Fig. 3 D), social media in disaster relief is the hot research topic in recent years. As a new communication technology, social media has become an essential channel for information dissemination during disaster relief (44). In disaster response, social media users collect data as sensors and then spread information through social media. In addition, social media such as Facebook and Twitter, have played an essential role in raising awareness, coordinating relief effort and collecting donations (44, 45). However, with the use of social media in disaster response, its defects have gradually attracted the attention of researchers. There are two significant challenges: there is no guarantee of the quality of information coming from social media and how to protect the privacy of social media users. Due to the characteristics of social media itself, the data from social media is much and complicated, among which there is false information. Thus, information filtering should be carried out before disaster assessment and decision-making based on this information to ensure the quality of information (45, 46). Also, the privacy of social media users should be paid attention to. Some studies show that social networking websites are at risk of revealing users’ information, and social media research often ignores this problem (46, 47).

Another significant result is that earthquakes, extreme weather and floods are the major natural disasters studied (n=143, 84.62%) (Fig. 4). These three kinds of disasters occur frequently and do serious harm. From 1990 to 2018, earthquakes, extreme weather and floods were reported 7,468 times, accounting for 79.23% of all disasters in that period (3). They caused 1.43 million deaths and 2.66 trillion economic losses, accounting for 86.24% and 90.14% of the total respectively. Therefore, the disaster management of earthquake, extreme weather and flood should be paid great attention by decision makers, practitioners and researchers.

Only 52 articles (34.32%) reported that ICTs were used within 72 h after the disaster (Table 7). As we all know, for disaster victims, they will have a great chance of survival, if they are rescued within the first 24 to 72 h (39). Therefore, rapid rescue after disasters is the first factor to reduce casualties. However, there is another problem that the application time of technology may not be consistent with the time to support decision-making. For example, remote sensing technology can acquire images of disaster areas in a short time after disasters, but the time for subsequent image processing may be long, so near real-time image processing technology is necessary (39).

What is worth our attention is that only five articles (2.96%) reported that ICTs can reduce casualties or economic losses in the disaster response phase. The former had less economic losses by comparing the people who used social media and those who did not during flood (30). Radar and radio can locate victims of Avalanches, shorting rescue time and reducing mortality (31–33). Telemedicine reduced the casualties of hurricane victims (34). However, two articles (1.18%) concluded that the rescue robot was ineffective in the mudslide rescue because of its poor water resistance (35, 36). Moreover, most studies (n=162, 95.86%) failed to pay attention to whether the technology has improved the relief outcome. In other words, the effectiveness of ICTs in disaster relief has not been systematically evaluated so far. The occurrence of disasters is unpredictable, so in the disaster response phase, responders including decision makers, rescue organizations and victims will face greater challenges than other disaster phases (48, 49). At present, most disaster relief operations, including the use of ICTs, are based on experience (50). However, the increase of disaster frequency and hazards and the scarcity of resources require more effective and efficient rescue operations (51). Performance evaluation can promote such improvement by evaluating the efficiency, effectiveness, responsiveness and flexibility of response measures (50). Therefore, the construction and implementation of ICTs performance evaluation framework during the rescue is the future research direction in this field. In addition, if there is a reporting guideline on ICTs application in disaster relief, it will be very helpful to evaluate the effectiveness of ICTs in the scenario of disaster rescue.

### Strengths and Limitations

As far as we know, this study is the first systematic review of ICTs application in natural disaster relief. However, limitations could not be avoided. First, the method of systematic review may not be able to review all literatures that meet the standards. In addition, we only included articles published in English, which might lead to language bias.

## Conclusion

The number of papers studying ICTs and disaster relief shows a general trend of growth since 1990. ICTs play vital roles in information dissemination, post-disaster image collection and damage assessment. In addition, ICTs have the potential to reduce casualties and economic losses, but some technologies are not applicable to all rescue scenarios. However, most studies failed to evaluate the effect of technology on the rescue. Therefore, the performance of ICTs needs to be systematically evaluated in order to better deploy ICTs and improve rescue activities. At the same time, a reporting guideline on ICTs and disaster relief is needed to guide researchers to standardize report in more detail.

## Ethical considerations

Ethical issues (Including plagiarism, informed consent, misconduct, data fabrication and/or falsification, double publication and/or submission, redundancy, etc.) have been completely observed by the authors.
